# Metabolic RNA Labeling and Translating Ribosome Affinity Purification for Measurement of Nascent RNA Translation

**DOI:** 10.21769/BioProtoc.5091

**Published:** 2024-10-20

**Authors:** Hirotatsu Imai, Akio Yamashita

**Affiliations:** Department of Investigative Medicine, University of the Ryukyus, Uehara 207, Okinawa, Japan

**Keywords:** Translating ribosome affinity purification, Metabolic RNA labeling, Transcription, Translation, Deep sequencing, 4-thiouridine, TRAP-seq

## Abstract

Regulation of gene expression in response to various biological processes, including extracellular stimulation and environmental adaptation, requires nascent mRNA synthesis and translation. Simultaneous analysis of the coordinated regulation of dynamic mRNA synthesis and translation using the same experiment remains a major challenge in the field. Here, we describe a step-by-step protocol for the simultaneous measurement of transcription of nascent mRNA and its translation at the gene level during the acute unfolded protein response (UPR) in HEK293 cells by combining 4-thiouridine metabolic mRNA labeling with translational ribosome affinity purification (TRAP) using a monoclonal antibody against evolutionarily conserved ribosomal P-stalk proteins (P-TRAP). Since P-TRAP captures full-length RNAs bound to ribosomes, it is compatible with 3′ mRNA-seq, which analyzes the uridine-rich 3′ UTRs of polyadenylated RNAs, allowing robust quantification of T>C conversions. Our nascent P-TRAP (nP-TRAP) method, in which P-TRAP is combined with metabolic mRNA labeling, can serve as a simple and powerful tool to analyze the coordinated regulation of transcription and translation of individual genes in cultured cells.

Key features

• Simple and retriable analysis of nascent mRNA synthesis and its translation in cultured cells

• Combination of 4-thiouridine metabolic RNA labeling with translating ribosome affinity purification (TRAP)

• Ribosomal P-stalk-mediated TRAP (P-TRAP) allows single-step and efficient purification of non-tagged ribosomes and translated mRNAs

## Graphical overview


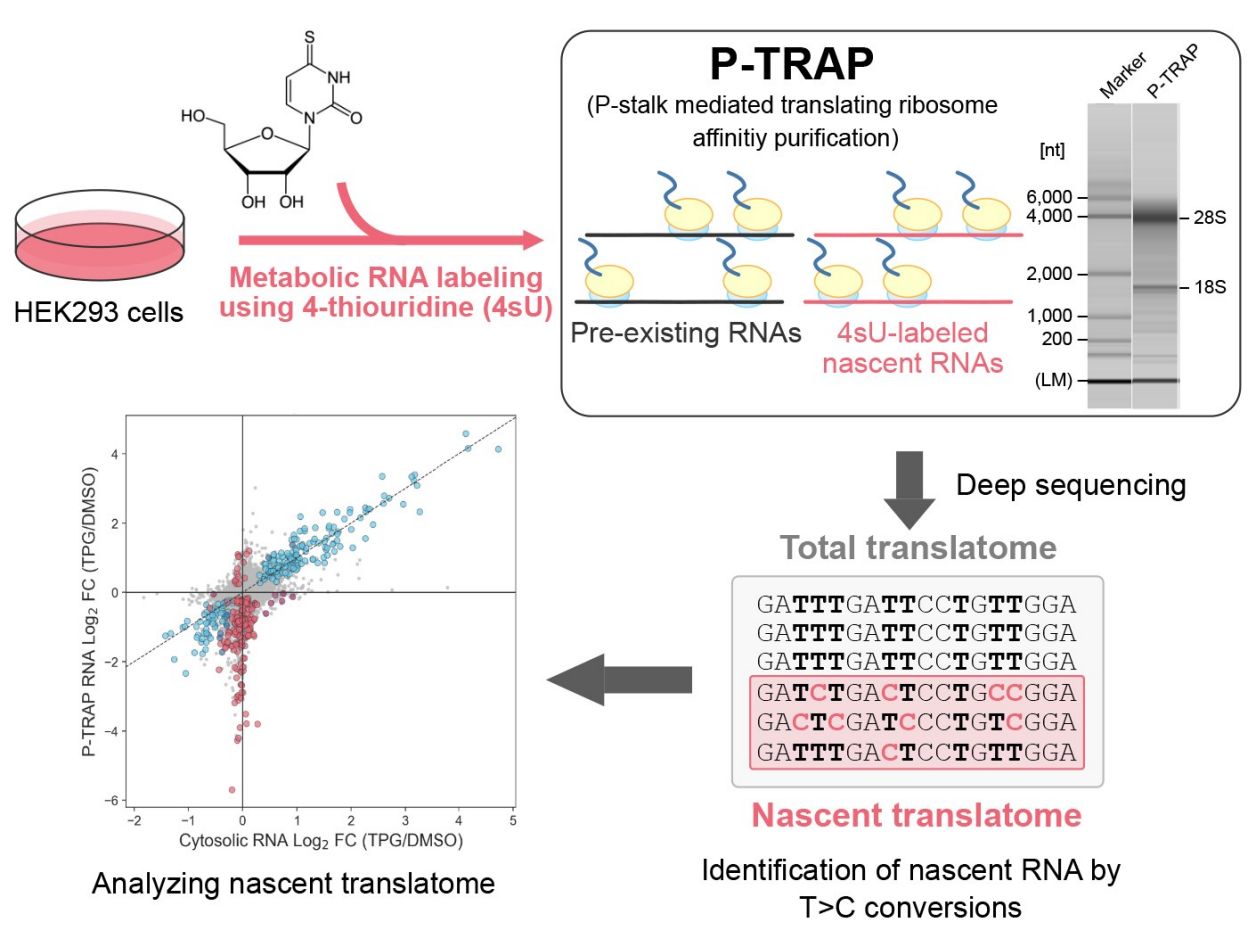


## Background

Regulation of gene expression plays a central role in many biological processes. The final output of a protein-coding gene, the amount of protein, is determined by several regulation processes: transcription and degradation of mRNA, translation of mRNA, and degradation of protein.

Metabolic RNA labeling techniques have been developed to analyze the dynamic transcription of nascent mRNA and its degradation. One of these labeling techniques is a thiol (SH)-linked alkylation for the metabolic sequencing of RNA (SLAMseq), in which RNAs are labeled with the uridine analog 4-thiouridine (4sU) [1]. 4sU is incorporated into nascent RNAs by RNA polymerase II and then alkylated during cDNA library preparation, allowing bioinformatic analysis to detect the specific T-to-C (T>C) conversion at the 4sU incorporation site in 4sU-labeled RNA. By focusing on sequence reads containing T>C conversions, the accumulation rate of nascent transcribed RNA after the addition of 4sU (progressive labeling technique) or the degradation rate of pre-4sU-labeled RNA after the addition of an excess amount of uridine (pulse-chase technique) can be analyzed [2,3].

In addition to mRNA transcription and its degradation, mRNA translation significantly influences the level of gene expression [4]. Currently, to analyze the level of translation, three different methods are employed utilizing next-generation sequencing techniques: polysome profiling, translating ribosome affinity purification (TRAP), and ribosome profiling (Ribo-seq). Polysome profiling fractionates full-length ribosome-bound RNAs using sucrose density gradient ultracentrifugation. TRAP also fractionates full-length ribosome-bound RNAs, but it relies on the immunoprecipitation of ribosomes. Ribo-seq fractionates ribosome-protected RNA fragments (RPFs) after sucrose density gradient via ultracentrifugation and RNase digestion. Each method has advantages and disadvantages and is used according to the experimental design and objectives (reviewed in [5]).

The most dominant regulation process that determines the protein level is different for each gene. Therefore, the simultaneous measurement of these regulation processes in a single experiment is advantageous to understand the regulatory mechanism of gene expression, which is still a major challenge in this field. Kawata et al. simultaneously evaluated the synthesis rate of nascent mRNA and the degradation rate of pre-existing mRNA by using two different metabolic labeling analogs, i.e., 5′-bromo-uridine (BrU) and 4sU [6]. Statistical approaches have also been developed to estimate both the synthesis rate of nascent mRNA and the degradation rate of pre-existing mRNA using only 4sU labeling [2,7]. Recently, Schott et al. developed nascent Ribo-seq (nRibo-seq), a combination of 4sU metabolic RNA labeling and Ribo-seq, which allows simultaneous measurement of nascent mRNA synthesis and its translation at the level of bulk or specific RNA groups [8]. Although nRibo-seq is a pioneering approach for measuring mRNA transcription and translation, Ribo-seq deals with the short length of RPFs, making the reliable quantification of 4sU incorporation for individual genes difficult [8].

Here, we describe a step-by-step protocol for the simultaneous measurement of transcription of nascent mRNA and its translation at the gene level in the acute unfolded protein response (UPR), in which transcription and translation are dynamically reprogrammed to reduce unfolded and misfolded proteins in the endoplasmic reticulum and restore protein homeostasis [9] in HEK293 cells by combining 4sU metabolic mRNA labeling with TRAP (originally reported in [10]). The use of a monoclonal antibody against the evolutionarily conserved ribosomal proteins P0, P1, and P2 (P-stalk) is responsible for the highly efficient purification of endogenous translating ribosomes without an affinity tag (e.g., FLAG-tag, His-tag, and SBP-tag), shortening the experimental period (termed as the P-TRAP method). Combining this P-TRAP with 4sU metabolic mRNA labeling and 3′ mRNA-Seq (which analyzes the uridine-rich 3′ UTR of polyadenylated RNAs) results in robust quantification of T>C conversion and reliable analysis of nascent mRNA transcription as well as its translation at the individual gene level. If the sensitivity of nascent RNA detection is sufficient, subtracting T>C reads from total reads will yield non-T>C reads that are mostly derived from pre-existing RNAs, contributing to further analysis of translation in not only nascent but also pre-existing RNAs. This simple and versatile nascent P-TRAP (nP-TRAP) method could enhance our understanding of the complex regulation of gene expression in eukaryotes.

## Materials and reagents

**Biological materials**

1. Flp-In^TM^ T-REX^TM^ 293 cell line (Thermo Fisher Scientific, catalog number: R78007)

**Reagents**

1. DMEM (high glucose) with L-Glutamine, phenol red and sodium pyruvate (FUJIFILM Wako, catalog number: 043-30085)
2. Penicillin-streptomycin mixed solution (Nacalai Tesque, catalog number: 09367-34)
3. Fetal bovine serum (FBS) (Cell Culture Bioscience, catalog number: 171012)
4. D-PBS(-) without Ca and Mg, liquid (Nacalai Tesque, catalog number: 14249-24)
5. Nuclease-free water (not DEPC-treated) (Thermo Fisher Scientific, catalog number: AM9937)
6. HEPES (Merck, catalog number: H3375-500G)
7. Tris (Nacalai Tesque, catalog number: 035401-25)
8. HCl (FUJIFILM, catalog number: 080-01066)
9. Glycine (FUJIFILM, catalog number: 077-00735)
10. MgCl_2_ (Nacalai Tesque, catalog number: 20908-65)
11. NaCl (FUJIFILM Wako, catalog number: 191-01665)
12. NaH_2_PO_4_·H_2_O (Nacalai Tesque, catalog number: 31719-05)
13. Na_2_HPO_4_·12H_2_O (FUJIFILM Wako, catalog number: 196-02835)
14. DTT (Roche, catalog number: 10708984001)
15. Triton X-100 (Nacalai Tesque, catalog number: 35501-15)
16. Tween 20 (FUJIFILM, catalog number: 167-11515)
17. SDS (SERVA, catalog number: 20765.02)
18. DMSO (FUJIFILM Wako, catalog number: 043-07211)
19. Glycerol (FUJIFILM Wako, catalog number: 075-00616)
20. Bromophenol blue (FUJIFILM Wako, catalog number: 021-02911)
21. BSA (Nacalai Tesque, catalog number: 01860-07)
22. Protease inhibitor cocktail for use with mammalian cell and tissue extracts (Nacalai Tesque, catalog number: 25955-11)
23. Phosphatase inhibitor cocktail (EDTA-free) (Nacalai Tesque, catalog number: 07575-51)
24. 4-thiouridine (4sU) (LKT Laboratory, catalog number: T2933)
25. Thapsigargin (Cayman Chemical, catalog number: 10522)
26. Cycloheximide (Nacalai Tesque, catalog number: 06741-91)
27. RNase A (100 mg/mL) (NIPPON GENE, catalog number: 318-06391)
28. RNasin^®^ Plus ribonuclease inhibitor (Promega, catalog number: N2611)
29. Dynabeads^TM^ protein G for immunoprecipitation (Thermo Fisher Scientific, catalog number: 10004D)
30. Anti-ribosomal proteins P0/P1/P2 mAb [9D5] (MBL, catalog number: RN004M)
31. Mouse IgG2a isotype control (Proteintech, catalog number: 65208-1-Ig)
32. Antibodies for western blotting:
  - Anti-ribosomal protein uL3 (GeneTex, catalog number: GTX114725)
  - Anti-ribosomal protein uS2 (GeneTex, catalog number: GTX114734)
  - Anti-PABP4 (BETHYL, catalog number: A301-467A), primary antibody
  - Anti-CBP80 (BETHYL, catalog number: A301-793A), primary antibody
  - Anti-eIF4A3 [11], primary antibody
  - Anti-GAPDH mAb-HRP-DirecT (MBL, catalog number: M171-7)
  - Anti-rabbit IgG, HRP-linked antibody (Cell Signaling Technology, catalog number: 7074S)
  - Anti-mouse IgG, HRP-linked antibody (Cell Signaling Technology, catalog number: 7076S)
33. ProClin^TM^ 950 (Merck, catalog number: 46878-U)
34. e-PAGEL 5%–20% 18-well precast mini gel (ATTO, catalog number: E-R520L)
35. Pre-stained XL-Ladder Broad (APRO, catalog number: SP-2110)
36. EzFastBlot Fast western blotting transfer buffer (ATTO, catalog number: AE-1465)
37. ECL select^TM^ western blotting detection reagent (Cytiva, catalog number: RPN2235)
38. ImmunoStar^®^ LD (FUJIFILM, catalog number: 296-69901)
39. ISOGEN II (NIPPON GENE, catalog number: 311-07361)
40. 2-Propanol (Nacalai Tesque, catalog number: 29113-95)
41. Ethanol (99.5) (Nacalai Tesque, catalog number: 14713-95)
42. Iodoacetamide, No-Weigh^TM^ format (Thermo Fisher Scientific, catalog number: A39271)
43. Sodium acetate nuclease and protease tested (Nacalai Tesque, catalog number: 31137-25)
44. 3 M NaOAc pH 5.2 (FUJIFILM Wako, catalog number: 316-90081)
45. 10 N NaOH (Nacalai Tesque, catalog number: 94611-45)
46. Glycogen solution (20 mg/mL) from Oyster, nuclease tested (Nacalai Tesque, catalog number: 17110-11)
47. TE buffer pH 8.0 (Nacalai Tesque, catalog number: 06890-54)
48. RNA 1000 reagent kit for MultiNA (SHIMADZU, catalog number: 292-27913-91)
49. SYBRTM Green II RNA gel stain, 10,000× concentrate in DMSO (Invitrogen, catalog number: S7568)
50. RNA 6000 ladder (Invitrogen, catalog number: AM7152)
51. Formamide (Nacalai Tesque, catalog number: 16229-95)
52. QuantSeq 3′ mRNA-Seq Library Prep Kit FWD for Illumina (Lexogen, catalog number: 192.14)
*Note: We used the currently unavailable catalog number 015.24. One may use 191.24 as an alternative.*
53. Qubit^®^ dsDNA HS Assay Kits (molecular probes life technologies, catalog number: Q32854)

**Solutions**

1. 20× TBS stock solution
2. 1 M Tris-HCl pH 7.5 stock solution
3. 1 M MgCl_2_ stock solution
4. 5 M NaCl stock solution
5. 1 M DTT stock solution
6. 1% SDS stock solution
7. Tris-glycine SDS running buffer (see Recipes)
8. 1 M HEPES-NaOH pH 7.5 stock solution (see Recipes)
9. 100 mg/mL cycloheximide (see Recipes)
10. 1% Triton X-100 lysis buffer (see Recipes)
11. Wash buffer (see Recipes)
12. SDS buffer (see Recipes)
13. 6× SDS-dye (see Recipes)
14. TBST (see Recipes)
15. 1% (w/v) BSA/TBST (see Recipes)
16. 200 mM 4-thiouridine (4sU) (see Recipes)
17. 1 mM Thapsigargin (TPG)
18. 1 M NaH_2_PO_4_ (see Recipes)
19. 1 M Na_2_HPO_4_ (see Recipes)
20. 0.5 M NaPO_4_ pH 8.0 (see Recipes)
21. 100 mM IAA (see Recipes)

**Recipes**

1. **Tris-glycine SDS running buffer (2,000 mL)**
  **Table:** 

Reagent	Final concentration	Quantity or Volume
Tris	25 mM	6 g
Glycine	192 mM	28.8 g
SDS	0.1% (w/v)	2 g
Deionized water	n/a	Up to 2,000 mL
*Note: Store at room temperature (RT).*
2. **1 M HEPES-NaOH pH 7.5 (1,000 mL)**
  **Table:** 

Reagent	Final concentration	Quantity or Volume
HEPES	1 M	238.3 g
10 N NaOH	n/a	To pH 7.5
Deionized water	n/a	Up to 1,000 mL
*Notes:*
  - *Prepare ~800 mL of deionized water in a suitable container. Add 238.3 g of HEPES to the solution. Adjust solution to the desired pH using 10 N NaOH. Add deionized water until the volume is 1,000 mL.*
  - *Store at 4 °C.*
3. **100 mg/mL cycloheximide (0.1 mL)**
  **Table:** 

Reagent	Final concentration	Quantity or Volume
Cycloheximide	100 mg/mL	10 mg
DMSO	n/a	0.1 mL
*Note: Prepare before use.*
4. **1% Triton X-100 lysis buffer (10 mL)**
  **Table:** 

Reagent	Final concentration	Quantity or Volume
1 M HEPES-NaOH pH 7.5	20 mM	0.2 mL
1 M MgCl_2_	2.5 mM	0.025 mL
5 M NaCl	150 mM	0.3 mL
Triton X-100	1% (v/v)	0.1 mL
1 M DTT	0.5 mM	5 μL
Cycloheximide (100 mg/mL)	100 μg/mL	10 μL
Protease inhibitor cocktail (100×)	1×	0.1 mL
Phosphatase inhibitor cocktail (100×)	1×	0.1 mL
Nuclease-free water	n/a	Up to 10 mL
*Notes:*
  - *Store at RT.*
  - *Add DTT, cycloheximide, protease inhibitor cocktail, and phosphatase inhibitor cocktail before use.*
5. **Wash buffer (50 mL)**
  **Table:** 

Reagent	Final concentration	Quantity or Volume
1 M HEPES-NaOH pH 7.5	20 mM	1 mL
1 M MgCl_2_	2.5 mM	0.125 mL
5 M NaCl	150 mM	1.5 mL
Tween 20	0.025% (v/v)	12.5 μL
Nuclease-free water	n/a	47.375 mL
*Note: Store at RT.*
6. **SDS buffer (10 mL)**
  **Table:** 

Reagent	Final concentration	Quantity or Volume
1 M HEPES-NaOH pH 7.5	20 mM	1 mL
1% SDS	1%	1 mL
Nuclease-free water	n/a	8 mL
*Note: Store at RT.*
7. **6× SDS-dye (10 mL)**
  **Table:** 

Reagent	Final concentration	Quantity or Volume
1 M Tris-HCl pH 7.5	0.3 M	3 mL
SDS	9% (w/v)	0.9 g
Glycerol	30% (v/v)	3 mL
1 M DTT	0.6 M	6 mL
Bromophenol blue	0.1% (w/v)	10 mg
Nuclease-free water	n/a	Up to 10 mL
*Note: Store at -20 °C.*
8. **TBST (2,000 mL)**
  **Table:** 

Reagent	Final concentration	Quantity or Volume
20× TBS	1×	100 mL
Tween 20	0.1% (v/v)	2 mL
Deionized water	n/a	Up to 2,000 mL
*Note: Store at RT.*
9. **1% BSA/TBST (50 mL)**
  **Table:** 

Reagent	Final concentration	Quantity or Volume
1× TBST	1×	50 mL
BSA	1% (w/v)	0.5 g
*Note: Prepare before use.*
10. **200 mM 4-thiouridine (0.5 mL)**
  **Table:** 

Reagent	Final concentration	Quantity or Volume
4-thiouridine (MW 260.27)	200 mM	26 mg
Nuclease-free water	n/a	0.5 mL
*Note: Store at -20 °C and protected from light.*
11. **1 mM Thapsigargin (1.5 mL)**
  **Table:** 

Reagent	Final concentration	Quantity or Volume
Thapsigargin (MW 650.8)	1 mM	1 mg
DMSO	n/a	1.536 mL
*Note: Dispense 20 μL each and store at -80 °C.*
12. **1 M NaH_2_PO_4_ (10 mL)**
  **Table:** 

Reagent	Final concentration	Quantity or Volume
NaH_2_PO_4_·H_2_O	1 M	1.38 g
Nuclease-free water	n/a	Up to 10 mL
*Note: Store at RT.*
13. **1 M Na_2_HPO_4_ (10 mL)**
  **Table:** 

Reagent	Final concentration	Quantity or Volume
Na_2_HPO_4_·12H_2_O	1 M	3.58 g
Nuclease-free water	n/a	Up to 10 mL
*Note: Store at RT.*
14. **0.5 M NaPO_4_ pH 8.0 (10 mL)**
  **Table:** 

Reagent	Final concentration	Quantity or Volume
1 M NaH_2_PO_4_	0.034 M	0.34 mL
1 M Na_2_HPO_4_	0.466 M	4.66 mL
Nuclease-free water	n/a	5 mL
*Note: Store at RT after 0.22 μm filtration.*
15. **100 mM IAA (0.5 mL)**
  **Table:** 

Reagent	Final concentration	Quantity or Volume
Iodoacetamide, No-Weigh^TM^	100 mM	9.3 mg (1 vial)
EtOH	n/a	0.5 mL
*Note: Prepare before use.*

**Laboratory supplies**

1. Cell culture 6-well plate (SPL Life science, catalog number: 30006)
2. Tissue culture dish Φ96 × 21 mm (10 cm) (TPP, catalog number: 93100)
3. Falcon^®^ 5 mL serological pipette (CORNING, catalog number: 357543)
4. Falcon^®^ 10 mL serological pipette (CORNING, catalog number: 357551)
5. FastGene^TM^ centrifuge tubes 15 mL (NIPPON Genetics, catalog number: FG400)
6. SuperClear^®^ centrifuge tubes 50 mL (Labcon, catalog number: 3181-345)
7. Round-bottom micro tube 1.7 mL (BIO-BIK, catalog number: RC-0170)
8. Siliconized microcentrifuge tube 1.5 mL (WATSON, catalog number: 131-615CH)
9. FastGene^TM^ 0.2 mL 8 strips PCR tube and FlatCap (NIPPON Genetics, catalog number: FG-028FC)
10. Long tip low adsorption 10 μL (BMBio ST, catalog number: BMSSCH0001)
11. FastGene^TM^ tip 200 μL (NIPPON Genetics, catalog number: FG-3001)
12. Long tip low adsorption 1,000 μL (BMBio ST, catalog number: BMSSCH0004)
13. Siliconized tip 1,000 μL (WATSON, catalog number: 121-814CH)
14. Immobilon^®^-P PVDF membrane (Millipore, catalog number: IPVH00010)
15. Chromatography paper (ATTO, catalog number: CB-20A)

## Equipment

1. PIPETMAN P-10 (Gilson, catalog number: F144802)
2. PIPETMAN P-20 (Gilson, catalog number: F123600)
3. PIPETMAN P-200 (Gilson, catalog number: F123601)
4. PIPETMAN P-1000 (Gilson, catalog number: F123602)
5. Pipette Mate NEO (NICHIRYO, catalog number: 00-PMNEO)
6. Bioclean Bench (PHCbi, catalog number: MCV-B161S)
7. CO_2_ incubator Prescyto MG-71C (TAITEC, catalog number: 0081460-000)
8. Inverted phase contrast microscope (OLYMPUS, catalog number: CK40)
9. Thermo Minder SDminiN (TITEC, catalog number: 0068750-000)
10. Aspirator (NISSIN, catalog number: NVP-11)
11. High-speed refrigerated micro centrifuge (TOMY, catalog number: MX-305)
12. Biomedical cooler, 4 °C (NIHON FREEZER, catalog number: UKS-5410DHC)
13. Biomedical freezer, -20 °C (PHCbi, catalog number: MDF-U539)
14. Ultra-low freezer, -80 °C (PHCbi, catalog number: MDF-DU702VHS1)
15. VORTEX-GENIE 2 Mixer (M&S Instruments, catalog number: SI-0286)
16. Ice On I (SK BIO International, catalog number: IO-1)
17. DynaMag^TM^-Spin Magnet (Invitrogen, catalog number: 12320D)
18. Miniature rocker shaker (NISSIN, catalog number: NX-12)
19. Rotary Mixer with Oshiri Penpen A type (NISSIN, catalog number: NRC-20D)
20. Hybridization rotator (NISSIN, catalog number: SN-06BN)
21. Dry thermos unit DTU-1CN (TITEC, catalog number: 0075930-000)
22. MiniSlab, AE-6401 Gel caster included (ATTO, catalog number: WSE-1165W)
23. HorizeBLOT 2M (ATTO, catalog number: WSE-4025M)
24. ImageQuant LAS 4000 mini (Cytiva)
25. NanoDrop 1000 (Thermo Fischer Scientific, catalog number: ND-1000)
26. Microchip electrophoresis system for DNA/RNA analysis MultiNA (SHIMADZU, catalog number: MCE-202)
27. Agencourt SPRIPlate 96R super magnet plate (Beckman Coulter, catalog number: A32782)
28. SpectraMax Paradigm multi-mode microplate reader (Molecular Devices)

## Software and datasets

1.SLAM-DUNK v.0.4.3 (https://github.com/t-neumann/slamdunk) [12]

2.R v.4.2.1 (https://www.r-project.org/)

3.Python v.3.7 ( https://www.python.org/)

4.DESeq2 v.1.3.8 (http://www.bioconductor.org/packages/release/bioc/html/DESeq2.html) [13]

5.deltaTE (https://github.com/SGDDNB/translational_regulation) [14]

6.ImageQuant^TM^ TL v.1.2 (Cytiva)

## Procedure

Before extracting ribosome-bound RNAs for preparing cDNA libraries, we recommend validating that P-TRAP works well on a small scale by western blot as described in Sections A and B. P-TRAP can be applied to other eukaryotic cell lines and animals. We have successfully applied P-TRAP to human A549 cells, mouse B16 cells, mouse MIN6 cells, mouse liver, budding yeast, nematode, and zebrafish.

- **Immunoprecipitation and detection of translating ribosomes using western blot analysis in HEK293 cells**
  1. Seed 2 × 10^5^ HEK293 cells in 2 mL of DMEM supplemented with 10% FBS, 100 U/mL penicillin, and 100 μg/mL streptomycin into a 6-well plate and incubate overnight in a humidified incubator with 5% CO_2_ at 37 °C. Use one well per sample.
  2. After 24 h, aspirate the medium from the plate and add 250 μL of freshly prepared ice-cold 1% Triton X-100 lysis buffer (see Recipes).
*Note: In this condition, the ER fraction is soluble, but the nuclear fraction is insoluble.*
  3. Gently shake the 6-well plate to ensure that the lysis buffer covers the entire surface of each well, transfer to a 1.5 mL tube using a micropipette, and centrifuge at 20,000× *g* for 10 min at 4 °C using a refrigerated centrifuge with a swing rotor.
*Notes:*
    - ***Work on ice to keep the 6-well plate and tubes cold.*** *As the sample warms up and translation proceeds, the ribosomes will dissociate from the mRNA.*
    - *No need to use a cell scraper.*
  4. Collect the supernatant in a 1.5 mL tube. Keep 10 μL of the supernatant in another 1.5 mL tube as input fraction for western blotting. Add 2 μL of 6× SDS-dye (see Recipes) to input fraction, incubate at 95 °C for 5 min, and store at -20 °C.
  5. Prepare the following 10× antibody solution with RNase A (Table 1).
    **Table 1.** 10× antibody solution with RNase A

Reagent	Concentration (10×)	Amount per 1 sample
1% Triton X-100 lysis buffer (freshly prepared)	n/a	18.8 μL
1 μg/mL anti-ribosomal proteins P0/P1/P2 mAb [9D5]	0.05 μg/μL	1 μL
RNase A (100 mg/mL)	1 mg/mL	0.2 μL

*Note: The Mouse IgG2a isotype control (from Proteintech) can be used as a negative control for the 9D5 antibody.*
  6. Add 20 μL of 10× antibody solution with RNase A to 180 μL of the supernatant in a new 1.5 mL tube (the final antibody solution will be 1×) and rotate at 4 °C for 60 min on an NRC-20D rotary mixer with an Oshiri Penpen A type.
*Note: The NRC-20D with an Oshiri Penpen A type is a rotary mixer with a tube tapping function. Tapping the tube during stirring prevents the beads from sticking to the bottom of the tube and improves the efficiency of immunoprecipitation. However, it is not essential.*
  7. Dispense 7 μL of Dynabeads^TM^ Protein G beads per sample into a new 1.5 mL tube and wash the beads by 200 μL with wash buffer (see Recipes). Insert the tube into the magnetic rack and remove the clear supernatant.
  8. Transfer 200 μL of sample solution to the Dynabeads tube and rotate at 4 °C for 40 min on an NRC-20D rotary mixer with an Oshiri Penpen A type.
  9. Place the tube in a magnetic rack and remove the clear supernatant. Keep 10 μL of the supernatant in another 1.5 mL tube as an unbound fraction for western blotting. Add 2 μL of 6× SDS-dye (see Recipes) to the unbound fraction, incubate at 95 °C for 5 min, and store at -20 °C. This fraction helps us to check the efficiency of immunoprecipitation.
  10. Wash the beads three times with 1 mL of wash buffer (see Recipes). Each time, resuspend the beads in wash buffer using a micropipette with a siliconized pipette tip and transfer to a new 1.5 mL siliconized tube to allow adsorption of non-specific binding proteins to the tip and tube. Place the tube in a magnetic rack and remove the clear supernatant (wash buffer).
*Note: The beads should be suspended in the wash buffer and the supernatant removed as rapidly as possible. Furthermore, care should be taken to prevent the beads from drying out during this process.*
  11. Add 30 μL of SDS buffer (see Recipes) to the beads and resuspend to elute the immunoprecipitants. Place the tube in a magnetic rack and transfer the clear supernatant to a new 1.5 mL tube as an eluted fraction for western blotting. Add 6 μL of 6× SDS-dye (see Recipes) to the eluted fraction, incubate at 95 °C for 5 min, and store at -20 °C.
- **Western blotting**
  1. Place the e-PAGEL 5%–20% precast gel (18 wells) in the WSE-1165W mini slab filled with Tris-Glycine SDS running buffer (see Recipes) and load 1.5 μL of molecular weight marker and 5 μL of each sample prepared in Section A. Run the gel at 180 V for 50–60 min at RT.
  2. After the gel runs, activate the PVDF membrane by soaking it in EtOH for a few seconds and incubate with 1× EzFastBlot transfer buffer until use.
  3. Transfer proteins from the gel to PVDF membranes under wet transfer conditions at 7 mA/cm^2^ (e.g., 441 mA for 6 cm × 9 cm PVDF membrane) for 15 min in the WSE-4025M HorizeBLOT at RT.
  4. Block the membrane with 10 mL of 1% BSA/TBST (see Recipes) for at least 30 min at RT with gentle agitation on the NX-12 rocker shaker (speed: 3).
  5. Prepare each of the primary antibody mix (Table 2) in 50 mL tubes. Each primary antibody is diluted in 2 mL of 1% BSA/TBST and stored at 4 °C until use.
    **Table 2.** Primary antibody mix

Catalog numbers	Protein	Molecular weight	Amount in 2 mL of 1% BSA/TBST	Dilution
MBL, RN004N	P0	36,000	1 μL	1/2,000
GeneTex, GTX114725	uL3	46,000	1 μL	1/2,000
GeneTex, GTX114734	uS2	31,000	1 μL	1/2,000
BETHYL, A301-467A	PABP4	70,000	2 μL	1/1,000
BETHYL, A301-793A	CBP80	95,000	2 μL	1/1,000
(Okada-Katsuhata et al. [11])	eIF4A3	51,000	0.1 μL	1/20,000

*Note: Primary antibodies can be reused. For long-term storage, add 0.2 μL of the ProClin^TM^ 950 to 2 mL of the solution (1/10,000 dilution) and store at 4 °C.*
  6. Place the membrane in 50 mL super-seal cap tubes containing each primary antibody solution with the transferred side facing the inside of the tube. Set the 50 mL tube to the SN-06BN hybridization rotator and incubate at 4 °C with rotation overnight.
*Note: If necessary, cut the membrane according to the molecular weight of the target protein.*
  7. Wash the membranes three times with 10 mL of TBST (see Recipes) for at least 10 min at RT with gentle agitation on the NX-12 rocker shaker (speed: 3).
  8. Prepare each of the secondary antibody mix (Table 3) in 50 mL tubes. Each secondary antibody is diluted in 2 mL of 1% BSA/TBST and stored at 4 °C until use.
    **Table 3.** Secondary antibody mix

Catalog numbers	Organisms	Amount in 2 mL of 1% BSA/TBST	Dilution
CST, 7076S	Mouse	0.4 μL	1/5,000
CST, 7074S	Rabbit	0.4 μL	1/5,000

*Note: Secondary antibodies should be freshly prepared.*
  9. Place the membrane in 50 mL super-seal cap tubes containing each secondary antibody solution with the transferred side facing the inside of the tube. Set the 50 mL tube to the SN-06BN hybridization rotator and incubate at RT for at least 30 min with rotation.
  10. Wash the membranes three times with 10 mL of TBST solution for at least 10 min at RT with gentle agitation on the NX-12 rocker shaker (speed: 3).
  11. Turn on and boot the ImageQuant LAS 4000 mini. Then, incubate the membranes with the appropriate HRP western blot substrate solution on plastic wrap. Place the membrane in the LAS 4000 mini chamber and acquire images (Figure 1).
*Notes:*
    - *HRP substrate solutions vary in signal intensity depending on the product. Select the most appropriate one according to the titer of the antibody. The exposure time is automatically determined by the ImageQuant^TM^ TL software and is typically in the range of 1–60 s. Longer exposure times may be required if the antibody is inactivated.*
    - *Invert the membrane back and forth several times (at least two times) to ensure that sufficient substrate solution is spread over the membrane.*
    - *Take a picture with bright light to save an image with the position of the molecular weight markers on the membrane. It helps in the identification of the bands of interest.*
    **Figure 1.**
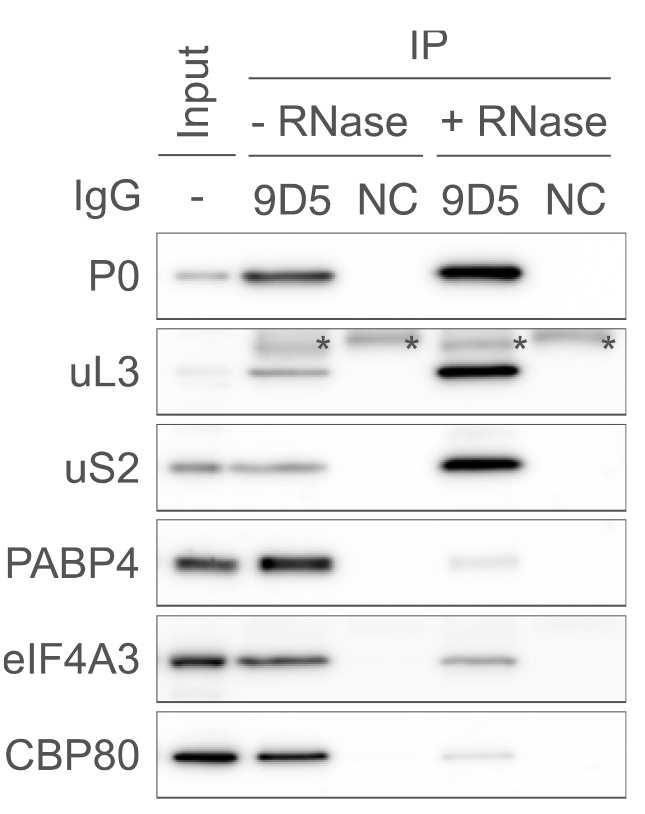
Typical results of western blot following P-TRAP. Immunoprecipitation of the endogenous ribosome and ribosome-bound RNA with the anti-ribosomal protein P0 antibody (9D5) or isotype control IgG (NC) from the cytosolic lysate of HEK293 cells in the presence or absence of RNase A, followed by western blotting using several primary antibodies (P0, uL3, uS2, PABP4, eIF4A3, and CBP80). The input contained 1% of the lysate used for immunoprecipitation. Asterisks (*) indicate non-specific signals from the antibodies (9D5 or NC). This figure is cited with modifications from the original publication [10].
- **4sU metabolic RNA labeling and extraction of cytosolic RNA and ribosome-bound RNA from HEK293 cells**
  This section describes the procedure for the extraction of cytosolic RNA and ribosome-bound RNA from HEK293 cells subjected to thapsigargin (TPG) to induce endoplasmic reticulum (ER) stress and 4sU for metabolic RNA labeling.
  1. Seed 1 × 10^6^ HEK293 cells in 10 mL of DMEM supplemented with 10% FBS, 100 U/mL penicillin, and 100 μg/mL streptomycin into a 10 cm dish and incubate overnight in a humidified incubator with 5% CO_2_ at 37 °C. Use one dish per sample.
*Note: Although we present the procedure using 10 cm dishes, scaling down is acceptable.*
  2. Twenty-four hours after seeding cells, prepare the following 4sU labeling mix (Table 4) for inducing ER stress and 4sU metabolic RNA labeling.
    **Table 4.** 4sU labeling mix

Reagent	Final conc. (in 10 cm dish)	Amount (per 1 sample)
Serum-free DMEM	n/a	180 μL
200 mM 4sU (see Recipes)	200 μM	10 μL
DMSO or 1 mM TPG (see Recipes)	0.1% (v/v) or 1 μM	10 μL
  3. Add labeling solution to the cultured medium (10 mL) and incubate for 3 h in a humidified incubator with 5% CO_2_ at 37 °C.
  4. Aspirate the medium from the plate and add 1 mL of freshly prepared ice-cold 1% Triton X-100 lysis buffer (see Recipes).
  5. Gently shake the 10 cm dish by hand to ensure that the lysis buffer covers the entire surface of each well, transfer to a 1.5 mL tube using a micropipette, and centrifuge at 20,000× *g* for 10 min at 4 °C, using a refrigerated centrifuge with a swing rotor.
*Notes:*
    - ***Work on ice to keep the 10 cm dishes and tubes cold.*** *As the sample warms up and translation proceeds, the ribosomes will dissociate from the mRNA.*
    - *No need to use a cell scraper.*
  6. Collect the supernatant in a 1.5 mL tube.
  7. Prepare the following 10× antibody mix with RNasin^®^ (Table 5).
    **Table 5.** 10× antibody solution with RNasin^®^

Reagent	Concentration (10×)	Amount per 1 sample
1% Triton X-100 lysis buffer (freshly prepared)	n/a	34 μL
1 μg/mL anti-ribosomal proteins P0/P1/P2 mAb [9D5]	0.2 μg/μL	4 μL
RNasin^®^ Plus ribonuclease inhibitor (40 units/μL)	10 units/μL	12 μL
  8. For cytosolic RNA preparation, transfer 200 μL of the supernatant in a new 1.5 mL tube. Add 500 μL of ISOGEN II supplemented with 1 mM DTT and mix well. Store the prepared sample solution at -20 °C.
  9. For ribosome-bound RNA preparation, transfer 450 μL of the supernatants in a new 1.5 mL tube. Add 50 μL of 10× antibody mix with RNasin to the supernatant (the final antibody solution will be 1×) and rotate at 4 °C for 60 min on an NRC-20D rotary mixer with an Oshiri Penpen A type.
*Note: The NRC-20D with an Oshiri Penpen A type is a rotary mixer with a tube tapping function. Tapping the tube during stirring prevents the beads from sticking to the bottom of the tube and improves the efficiency of immunoprecipitation. However, it is not essential.*
  10. Dispense 28 μL of Dynabeads^TM^ Protein G beads per sample into a new 1.5 mL tube and wash the beads with 1 mL of wash buffer (see Recipes). Insert the tube into the magnetic rack and remove the clear supernatant.
  11. Transfer all of the sample solution to the Dynabeads tube and rotate at 4 °C for 40 min on an NRC-20D rotary mixer with an Oshiri Penpen A type.
  12. Place the tube in a magnetic rack, remove the clear supernatant, and wash the beads three times with 1 mL of wash buffer (see Recipes). Each time, resuspend the beads in wash buffer using a micropipette with a siliconized pipette tip and transfer to a new 1.5 mL siliconized tube to allow adsorption of non-specific binding proteins to the tip and tube. Place the tube in a magnetic rack and remove the clear supernatant (wash buffer).
*Note: The beads should be suspended in the wash buffer and the supernatant removed as rapidly as possible. Furthermore, care should be taken to prevent the beads from drying out during this process.*
  13. Add 500 μL of ISOGEN II supplemented with 1 mM DTT to the beads and resuspend to elute the immunoprecipitants. Place the tube in a magnetic rack and transfer the clear supernatant to a new 1.5 mL tube. Add 200 μL of nuclease-free water supplemented with 1 mM DTT to the transferred ISOGEN II solution and mix well. The prepared sample solution is stored at -20 °C.
- **RNA purification and alkylation**
*Note: The initial steps of RNA purification and alkylation* ***must be performed*** *in the dark or protected from (white) light (e.g., by keeping samples covered, wrapping all tubes in aluminum foil, or working under red light).*
  1. Incubate the RNA samples (previously stored at -20 °C) at 37 °C for 5 min.
  2. Vortex for 15 s. Stand tubes at RT for 10 min and centrifuge at 20,000× *g* for 10 min at RT with a swing rotor.
  3. Transfer 500 μL of supernatants to the new 1.5 mL tubes and add 500 μL of 2-propanol supplemented with 1 mM DTT.
  4. Vortex for 15 s and centrifuge at 20,000× *g* for 15 min at RT with a swing rotor.
  5. Discard the supernatants by decantation, add 500 μL of 70% EtOH supplemented with 1 mM DTT, and centrifuge at 20,000× *g* for 3 min at RT with a swing rotor.
  6. Repeat step D5 once.
  7. Discard the supernatants by micropipetting.
  8. Add 15 μL of nuclease-free water supplemented with 1 mM DTT to the RNA pellet and resuspend.
  9. Measure RNA concentration by nanodrop.
*Note: To assess the purity of RNA, it is recommended to check the ratio of absorbance at 260 and 280 nm (a ratio of ~2.0 is acceptable).*
  10. Prepare the following DMSO/NaPO_4_ solution (Table 6).
    **Table 6.** DMSO/NaPO_4_ solution

Reagent	Concentration	Amount per 1 sample
DMSO	n/a	25 μL
0.5 M NaPO_4_ pH 8.0 (see Recipes)	83 mM	5 μL
*Notes: NaOP_4_ may form aggregates when added to DMSO. Prepare extra volume of DMSO/NaPO_4_ solution in a 1.5 mL tube (e.g., if you have 12 samples, prepare solution for 16 samples), centrifuge the mixed solution, and transfer only the clear supernatant to a new 1.5 mL tube. Use the supernatant for the following steps.*
  11. Prepare the following 4sU alkylation mix (Table 7).
    **Table 7.** 4sU alkylation mix

Reagent	Concentration	Amount per 1 sample
Sample RNA	≤ 100 ng/μL	X μL (≤ 5 μg)
Nuclease-free water	n/a	15–X μL
IAA (100 mM) (see Recipes)	10 mM	5 μL
DMSO/NaPO_4_ solution	n/a	30 μL
*Notes:*
    - *The volume of input RNA should be less than 5 μg.*
    - *Use only freshly prepared IAA.*
  12. Incubate at 50 °C for 15 min with the DTU-1CN Dry Thermal Unit.
  13. Add 1 μL of 1 M DTT, 1 μL of 20 mg/mg glycogen, 5 μL of 3 M NaOAc, and 125 μL of EtOH to each sample.
*Note: After this step, exposure to light is possible.*
  14. Vortex for 15 s and centrifuge at 20,000× *g* for 30 min at 4 °C with a swing rotor.
  15. Discard the supernatants by decantation, add 1 mL of 70% EtOH, and centrifuge at 20,000× *g* for 3 min at 4 °C with a swing rotor.
  16. Discard the supernatants by decantation, add 1 mL of 70% EtOH, and centrifuge at 20,000× *g* for 3 min at RT with a swing rotor.
  17. Discard the supernatants by micropipetting.
  18. Add 25 μL of nuclease-free water to the pellet and resuspend.
*Note: If the input RNA is less than 5 μg, the volume of nuclease-free water can be reduced (e.g., suspend the pellet in 12.5 μL of nuclease-free water if the input RNA is 2.5 μg).*
  19. Measure RNA concentration by nanodrop. Typical RNA concentrations are 100–200 ng/μL when using 5 μg of RNA as input.
*Note: To assess the purity of RNA, it is recommended to check the ratio of absorbance at 260 nm and 280 nm (a ratio of ~2.0 is acceptable).*
  20. Assess the quality of the RNA with the fragment analyzer MultiNA.
*Note: Instead of MultiNA, Bioanalyzer, TapeStation, or electrophoresis with denaturing TBE-agarose gel can be used.*
    - Bring separation buffer and marker solution (reagents from the RNA 1000 kit), SYBR Green II stock solution, and RNA 6000 ladder to RT.
    - Dilute SYBR Green II stock solution 100-fold with TE buffer.
    - Prepare the required volume of the following MultiNA buffer solution (Table 8) in a 5 mL screw cap tube (included in the RNA 1000 kit).
*Note: The required volume depends on the sample number. Prepare two more samples for the blank tube and RNA 6000 ladder.*
      **Table 8.** MultiNA buffer solution

Reagent	Amount for 1 sample	Amount for 20 samples
Separation buffer (a reagent of the RNA 1000 kit)	63.2 μL	1,264 μL
1/100 diluted SYBR Green II	0.8 μL	16 μL
Formamide	16 μL	320 μL
    - Prepare the following blank (Table 9), RNA 6000 ladder (Table 10), and RNA samples (Table 11) in 0.2 mL PCR tubes for the MultiNA run.
      **Table 9.** Blank

Reagent	Amount
RNA marker solution (a reagent of the RNA 1000 kit)	5 μL
RNA storage solution (a reagent of the RNA 1000 kit)	5 μL
      **Table 10.** RNA 6000 ladder

Reagent	Amount
RNA marker solution (a reagent of the RNA 1000 kit)	5 μL
RNA storage solution (a reagent of the RNA 1000 kit)	4 μL
RNA 6000 ladder	1 μL
      **Table 11.** RNA sample

Reagent	Amount (for 1 sample)
RNA marker solution (a reagent of the RNA 1000 kit)	5 μL
RNA storage solution (a reagent of the RNA 1000 kit)	4 μL
Sample RNA	1 μL
  21. Incubate at 65 °C for 5 min in a thermal cycler and immediately place on ice.
  22. Set the 5 mL tube (MultiNA buffer solution) and the 0.2 mL PCR tubes (blank, RNA 6000 ladder, and RNA samples) on MultiNA and start the run with MultiNA Control Software (Figure 2).
    **Figure 2.**
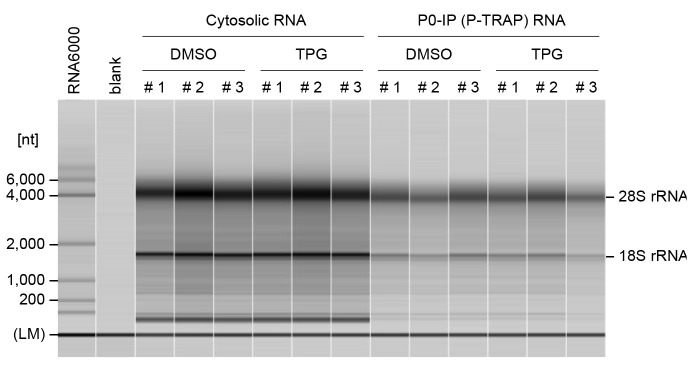
Quality control of RNA samples using a fragment analyzer. Representative capillary electrophoresis profiles for cytosolic RNAs and immunoprecipitated RNAs from DMSO- or TPG-treated HEK293 cells were analyzed using the MultiNA. Three independent experiments were performed for each condition (#1, #2, and #3). The lower marker (LM) indicates internal standards (25 nt). RNA ladder (Agilent RNA 6000 Pico Kit) was used as a size marker. The MultiNA Viewer software automatically assesses RNA size and concentration. RNA quality is assessed by the ratio of 28S to 18S rRNA.
- **Sequencing**
  1. Prepare the sequencing library using the QuantSeq 3′ mRNA-Seq Library Prep Kit FWD for Illumina, according to the manufacturer’s instructions. Use 500 ng of RNA as an input.
  2. Assess the quantity of the libraries with the Qubit^TM^ dsDNA Quantification kit.
    - Prepare the required volume of the following Qubit working solution (Table 12) in a 1.5 mL tube.
*Note: The required volume depends upon the sample number. Prepare 2 more samples for standards.*
      **Table 12.** Qubit working solution

Reagent	Amount for 1 sample	Amount for 20 samples
Qubit dsDNS HS Reagent (Component A)	50 μL	1,000 μL
Qubit dsDNS HS Reagent (Component B)	0.25 μL	5 μL
    - Mix the working solution with standards or samples (Table 13) in a 96-well black plate.
*Note: Pipetting of samples should be done gently to avoid bubble formation.*
      **Table 13.** Qubit working solution with standards or samples

Reagent	Amount for 1 sample
Qubit working solution (Table 12)	50 μL
Qubit dsDNS HS Standard (Component C or D) or cDNA sample	1 μL
    - Measure fluorescence using the SpectraMax Paradigm multi-mode microplate reader. The excitation wavelength is 485 nm and the emission wavelength is 530 nm. Measure each plate at least three times to check the stability of the fluorescence in each well.
    - Determine the concentration of each library and pool them in an equimolar ratio in a 1.5 mL tube for multiplex sequencing.
  3. Sequence samples on Illumina HiSeq system. Sequence so that there are at least 1 million counted T>C reads per sample (of course, the more the better).
*Notes:*
    - *We recommend longer sequencing read lengths (e.g., ≥ 150 bp) to increase the sensitivity for detecting T>C conversions in bioinformatic analysis.*
    - *If you run the sequence in pair-end mode, you will get two fastq files, read 1 (FWD) and read 2 (REV). In this case, use only read 1 (FWD) for analysis, because read 2 (REV) will start sequencing with the poly(T) strand on the 3′ side and sequence through the homopolymer strand, reducing the quality of the sequence reads.*
    - *Support for the Illumina HiSeq system is being phased out. It is recommended to use newer platforms such as the NovaSeq system.*

## Data analysis

We performed all sequencing data analysis steps under Ubuntu 20.04 LTS. Total and nascent RNAs were quantified using SLAM-DUNK, a pipeline for SLAMseq data analysis.

1. Make sure that SLAM-DUNK is installed on your Linux system. If not, install the latest version of SLAM-DUNK according to the document (https://t-neumann.github.io/slamdunk/index.html).
2. Prepare genome reference. For example, human reference FASTA files are prepared as follows:
  ```bash
  Wget http://ftp.ebi.ac.uk/pub/databases/gencode/Gencode_human/latest_release/GRCh38.p13.genome.fa.gz
  gunzip GRCh38.p13.genome.fa.gz
  ```
3. Prepare a BED file with 3′ UTR coordinates. This can be downloaded from the UCSF Table Browser (https://genome.ucsc.edu/cgi-bin/hgTables). Alternatively, you can create a BED file from the annotation file as follows, using the Python script “create_bed.py” provided as a supplemental file.
*Note: This Python script requires “pandas” and “gffpandas”. Place the annotation file and “create_bed.py” in the same directory and run it.*
  ```bash
  wget https://ftp.ebi.ac.uk/pub/databases/gencode/Gencode_human/release_41/gencode.v41.annotation.gff3.gz
  gunzip ge ncode.v41.annotation.gff3.gz
  python generate_BED.py
  ```
4. Run “slamdunk”. Here, 12 bases from the 5′ end were trimmed (--trim-5p 12), and then five or more consecutive adenines from the 3′ end were considered the remaining poly(A) tail and removed (--max-polya 4). Up to 100 regions with multiple mapped reads were allowed (--topn 100). For details on detecting T>C conversions, refer to the original paper [12].
  ```bash
  slamdunk map --trim-5p 12 --max-polya 4 --topn 100 --skip-sam --reference <reference fasta> --outputDir slamdunk/map <path to fastq file>
  slamdunk filter --outputDir slamdunk/filter slamdunk/map/*.bam
  slamdunk snp --outputDir slamdunk/snp --reference <path to reference fasta> slamdunk/filter/*.bam
  slamdunk count --reference <path to reference fasta> --bed <path to bed file> --outputDir slamdunk/count --snp-directory slamdunk/snp slamdunk/filter/*.bam
  ```
*Note: It is recommended that FASTQ files be trimmed and quality checked with a trimming tool, such as fastp [15], and a quality control tool, such as FastQC [16], before running “slamdunk”. In addition, tools such as MultiQC [17] can be used to summarize various parameters such as mapping quality and T>C conversion rate to obtain reliable results.*
5. Perform differential gene expression analysis using DESeq2 and differential transcription and translation analysis using the deltaTE method for nascent RNA (Figure 3).
*Note: For DESeq2 and deltaTE analyses, use the ReadCount (total read count) and TcReadCount (T>C read count) columns of the tcount file (output from “slamdunk count”) as input. To analyze the TcReadCount column with the DESeq2 and deltaTE method, calculate global normalization factors using the ReadCount column.*
  **Figure 3.**
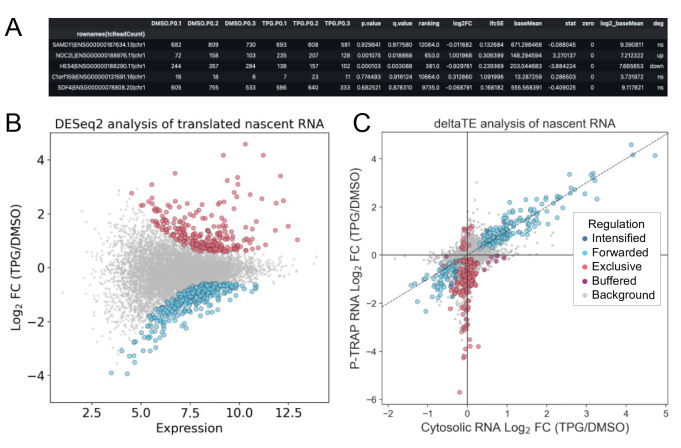
Differentially expressed and translated nascent RNA analysis using DESeq2 and deltaTE. Result table (first five rows) (A) and MA plot (B) of nascent RNA obtained by P-TRAP-seq from TPG-treated vs. DMSO-treated HEK293 cells. Results from DESeq2 using the ReadCount and TcReadCount columns as inputs in the "slamdunk" output file are plotted. Differentially expressed genes (adjusted p-value < 0.05 and log2 fold change > |1.5|) are highlighted in red (up-regulated) or blue (down-regulated). (C) Fold change of nascent RNAs in cytosolic RNA-seq and P-TRAP-seq data at the level of the individual genes is analyzed by the deltaTE method using the ReadCount and TcReadCount columns as inputs in the "slamdunk" output file (FDR < 0.05 and log2 fold change > |0|). Regulation modes categorized by deltaTE analysis as described [14]. [Translationally forwarded genes (cyan), intensified genes (blue), exclusive genes (red), and buffered genes (purple) are highlighted.] These figures are cited with modifications from the original publication [10].
6. To analyze the translation of pre-existing RNA, calculate the non-T>C read count by subtracting the T>C read count from the total read count.
*Notes:*
  - *The non-T>C reads are expected to include some reads that are derived from nascent RNAs but do not contain T>C conversion. If the proportion of non-T>C reads derived from these nascent RNAs is high, the estimation of the proportion of pre-existing RNAs based on the subtracted read counts will be inaccurate. Therefore, it is recommended to evaluate the sensitivity of the detection of T>C reads under each experimental condition. For example, focus on genes (CHAC1, DDIT3, and HERPUD1 in the UPR, etc.) that are known to be significantly transcribed after the addition of TPG, an inducer of ER stress. This is because most of the reads from these genes are likely to be derived from nascent RNAs. By calculating the proportion of reads detected as T>C reads out of all these reads, the sensitivity of the detection of T>C reads is improved (Figure 4).*
  - *To analyze the non-T>C read count with the DESeq2 and deltaTE method, calculate global normalization factors using the total read count column.*
7. For further analysis, output of DESeq2 (e.g., Log2FC) can be used to generate a cumulative curve to analyze the expression of nascent and pre-existing RNAs in specific gene categories (Figure 5).
  **Figure 4.**
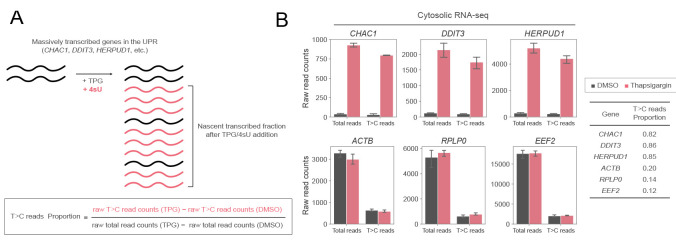
Evaluation of the detection sensitivity of T>C reads under each experimental condition. A. Schematic representation of nascent RNA synthesis of massively transcribed genes in the UPR and its 4sU incorporation. A certain percentage of RNAs may not be labeled by 4sU even though they are nascently transcribed. The proportion of 4sU labeling in RNA that is transcribed after thapsigargin treatment is used to assess detection sensitivity. B. Raw total read counts and raw T>C read counts of selected genes in cytosolic RNA-seq in response to DMSO (black) or thapsigargin (TPG) (red) treatment. The means and standard deviations of three replicates are shown. The proportion of T>C reads out of the total reads increased by TPG treatment for stress-induced genes (*CHAC1, DDIT3*, and *HERPUD1*) but not control genes (*ACTB, RPLP0*, and *EEF2*). These figures are cited with modifications from the original publication [10].
  **Figure 5.**
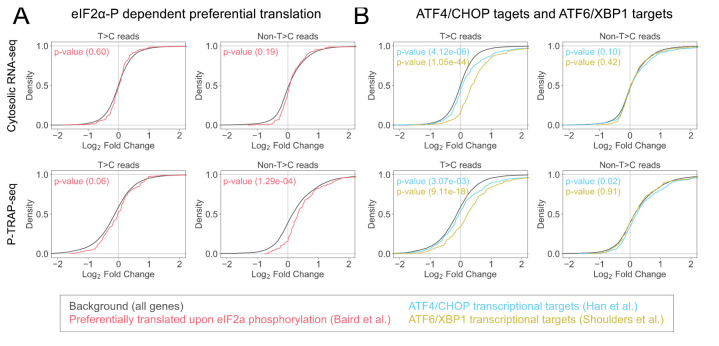
Categorical analysis of the gene expression of the nascent and pre-existing RNAs. Cumulative distributions of the Log2FC calculated by DESeq2 of specific gene categories, preferentially translated by polysome upon eIF2α phosphorylation [18] (A) and ATF6/XBP1 transcriptional targets [19,20] (B) compared to all genes, together with p-values of Mann–Whitney U test. These figures are cited with modifications from the original publication [10].

## Validation of protocol

This protocol or parts of it has been used and validated in the following research article(s):

- Imai et al. [10]. Simultaneous measurement of nascent transcriptome and translatome using 4-thiouridine metabolic RNA labeling and translating ribosome affinity purification. *Nucleic Acids Res*.

## Supplementary information

The following supporting information can be downloaded here:

1. gencode.v41.customized.canonical.bed
2. generate_bed.py
